# Pancreatic Hydatid Cyst Misdiagnosed as Mucinous Cystadenoma: CT and MRI Findings

**DOI:** 10.3390/medicina56030124

**Published:** 2020-03-13

**Authors:** Milica Mitrovic, Boris Tadic, Jelena Kovac, Nikola Grubor, Vladimir Milosavljevic, Aleksandra Jankovic, Igor Khatkov, Dejan Radenkovic, Slavko Matic

**Affiliations:** 1 Center for Radiology and Magnetic Resonance Imaging, Clinical Centre of Serbia, Pasterova No.2, 11000 Belgrade, Serbia; dr_milica@yahoo.com (M.M.); jelenadjokickovac@gmail.com (J.K.); jankovicm.alex@gmail.com (A.J.); 2 Department for HPB Surgery, Clinic for Digestive Surgery, Clinical Centre of Serbia, Koste Todorovica Street, No. 6, 11000 Belgrade, Serbia; tadicboris@yahoo.com (B.T.); n.grubor@yahoo.com (N.G.); dejanr09@yahoo.com (D.R.); 3 Department for Surgery, Faculty of Medicine, University of Belgrade, Dr Subotica No. 8, 11000 Belgrade, Serbia; 4 Department for Radiology, Faculty of Medicine, University of Belgrade, Dr Subotica No. 8, 11000 Belgrade, Serbia; 5 Gracia Medica Polyclinic, Koce Popovica Street, No. 9, 11000 Belgrade, Serbia; milosavljevicvladimir10@gmail.com; 6 Moscow Clinical Scientific Center, Enthuziastov Shosse, 86, 111123 Moscow, Russia; i.hatkov@mknc.ru

**Keywords:** hydatid cyst, *Echinococcus granulosus*, pancreas, laparoscopy, pancreatic cyst, imaging, distal pancreatectomy

## Abstract

Isolated hydatid cysts of the pancreas are rare lesions, even in endemic regions. In this report, we present the case of a 76-year-old patient who was admitted to our clinic with a diagnosis of a cystic lesion in the tail of the pancreas. On preoperative computed tomography (CT) and magnetic resonance (MR) examination, the cyst was characterized as a mucinous cystadenoma. A laparoscopic distal pancreatectomy followed. A histopathological examination revealed a large hydatid cyst in the tail of the pancreas.

## 1. Introduction

**Figure 1:**
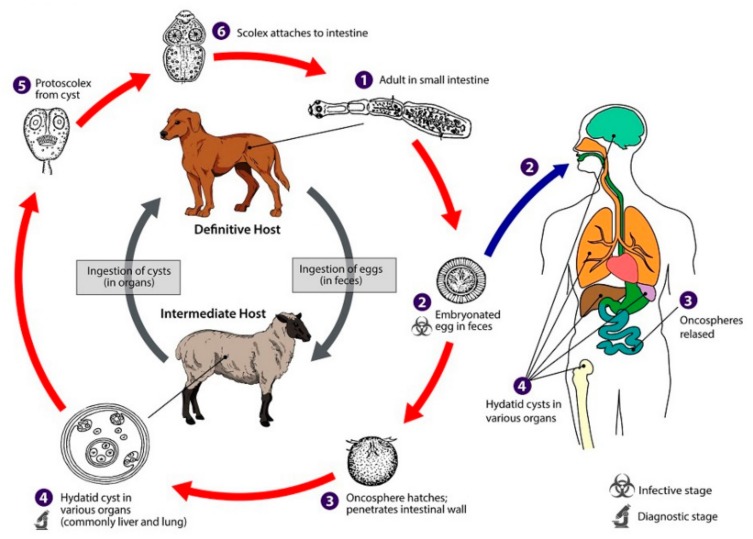
Life cycle of *Echinococcus granulosus* [4].

Hydatid disease is a zoonosis caused by the adult or larval stage of *Echinococcus granulosus* and is a common condition in cattle-raising countries [1,2]. Dogs are the definitive host for these tiny cestodes, while sheep and goats are intermediate hosts. The life cycle of *Echinococcus granulosus* develops in dogs and other canids, which harbor the adult tapeworm in the intestine. Humans, as intermediate hosts, are infected accidentally after the ingestion of food contaminated with parasite eggs (Figure 1). Once eggs are ingested by the intermediate host, the oncosphere affects the liver and other organs after penetrating the small intestine wall. This process leads to primary echinococcosis. Secondary echinococcosis results from the spillage of protoscoleces, or small daughter cysts, from the ruptured original cyst [3].

Although hydatid cysts can be found in any organ of the body, the most common sites of involvement are the liver, found in 50–77% cases, followed by lungs (15–47%), spleen (0.5–8%), and kidneys (2–4%). Primary pancreatic hydatid disease is extremely rare (0.14–2%) and occurs mainly by hematogenous dissemination [1]. Clinical symptoms depend on the size and localization of the lesion. Pancreatic hydatid cysts are usually solitary and mainly localized in the head (57%) and the body of the pancreas (24–34%). The pancreatic tail is the rarest localization (16–19%) [5]. This study was approved by the Ethics Committee of the Clinical Centre of Serbia No. 589/5 (date of approval 18.12.2019). Written informed consent was obtained from the patient.

## 2. Case Report

A 76-year-old woman was admitted to the clinic for digestive surgery on 17th December 2018 due to persistent mild nausea and chronic dull abdominal pain. A few days earlier, an abdominal ultrasound examination performed in an outdoor hospital revealed a large cystic mass engaging the pancreatic body and tail. The main findings of the initial physical examination were the presence of a palpable, painless mass in the left upper quadrant of the abdomen. Baseline hematological and biochemical investigations, including serum amylase and lipase, were within normal limits. Tumor markers were all within normal values (Carbohydrate antigen (CA) 19-9 2.06 U/mL, alpha-fetoprotein (AFP) 1.8 IU/mL, CA 125 14 U/mL, CA 15-3 21 U/mL, CA 72-4 5.9 kU/mL). In her previous medical documentation, we found that the patient had been treated for hypertension with a combination of an Angiotensin-converting enzyme (ACE) inhibitor and a diuretic. There were no data about previous attacks of acute pancreatitis.

**Figure 2:**
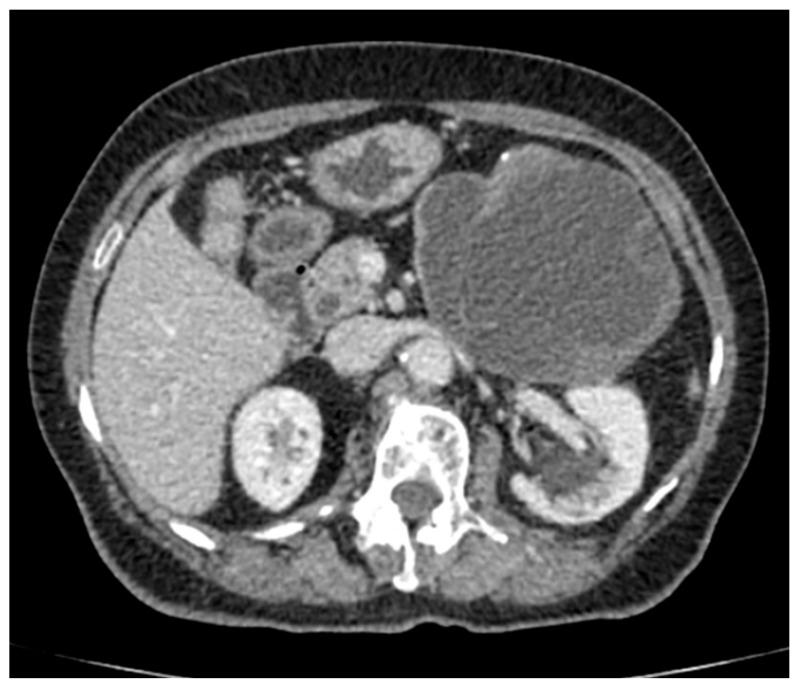
Axial section contrast-enhanced abdominal computed tomography (CT) shows a well-defined round to oval cystic lesion 116 × 100 mm large in the tail of the pancreas, causing compression of the stomach, with well-defined peripherally enhancing margins, and an irregularly thickened wall. Discrete internal septation and peripheral microcalcifications are also detected.

**Figure 3:**
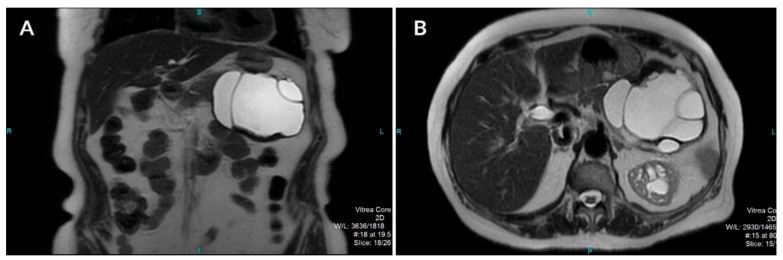
Coronal (**A**) and axial (**B**) T2-weighted images show a well-defined cystic lesion in the tail of the pancreas with high-signal-intensity inner loculi and a thick, complete hypointense rim with internal membranes.

**Figure 4:**
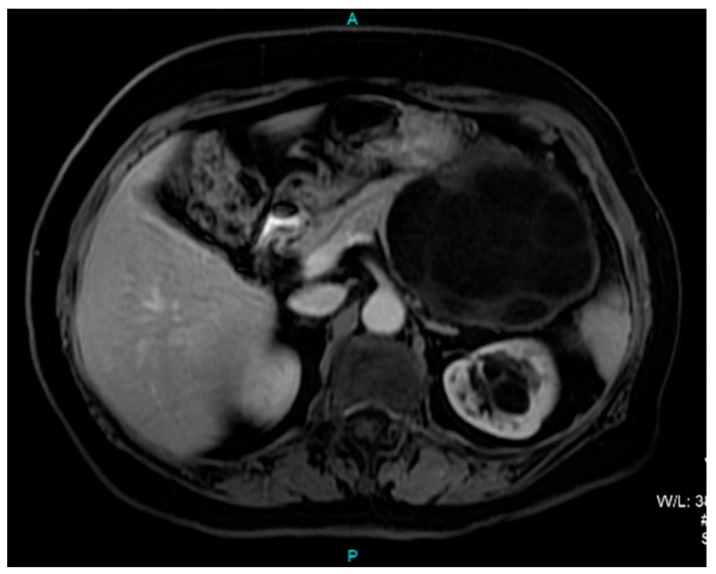
Axial T1-weighted postcontrast MRI image shows a predominantly hypointense cystic lesion in the region of the pancreatic tail without any solid component.

A computed tomography (CT) scan of the abdomen showed a well-defined macrocystic lesion in the tail of the pancreas, measuring approximately 11 cm, with thin internal septations and discrete peripheral linear wall calcification (Figure 2). Further magnetic resonance imaging (MRI) examination demonstrated the high signal intensity of the multilocular cystic lesion on the T2-weighted image, with an irregularly thickened wall (Figure 3). There was no detection of any solid component (Figure 4). No communication with the main pancreatic duct was seen. Moreover, a few internal septations were without a specific pattern. All these findings were suggestive of a diagnosis of mucinous cystadenoma. Additionally, an endoscopic ultrasound examination confirmed the MRI findings. Chests X-ray findings, routinely performed as part of a preoperative evaluation, were also unremarkable.

**Figure 5:**
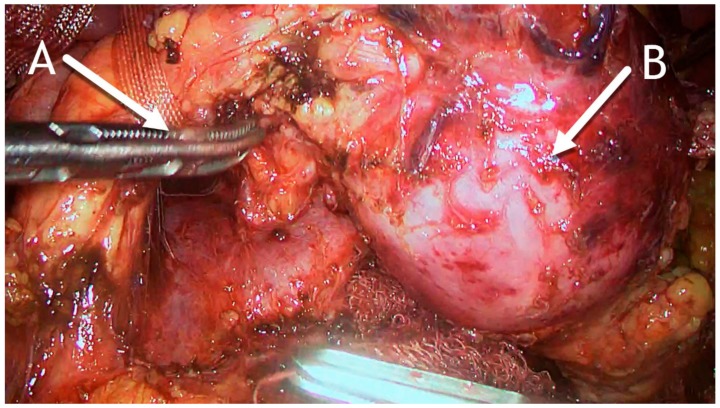
Intraoperative photo. (**A**) Pancreas dissected to expose the superior mesenteric vein and pulled up with tape. (**B**) Hydatid cyst in the distal part of the pancreas.

Considering the radiological findings, we opted for a laparoscopic distal pancreatectomy. After the gastrocolic ligament was transected, a large tumorous mass engaging the body and tail of the pancreas was found. A tunnel between the superior mesenteric vein (SMV) and the pancreas was made. Resection was performed at the level of the SMV using an Endo-GIA stapler (Figure 5). A specimen bag was inserted, and the resected part of the pancreas was placed in it. We applied a wound retractor to the widest port incision to avoid trauma to the surgical specimen and to prevent contact with the abdominal wall. Finally, the specimen was removed through it.

**Figure 6:**
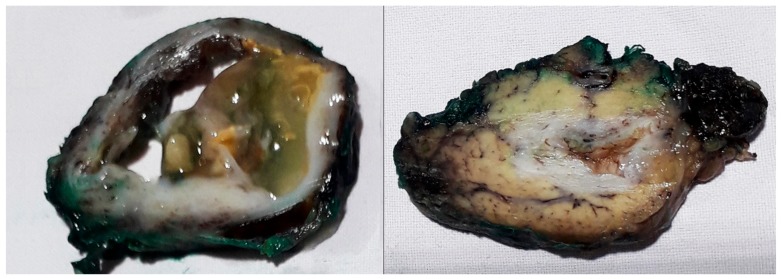
Specimen of the hydatid cyst showing a multiloculated cyst on the cut section.

**Figure 7:**
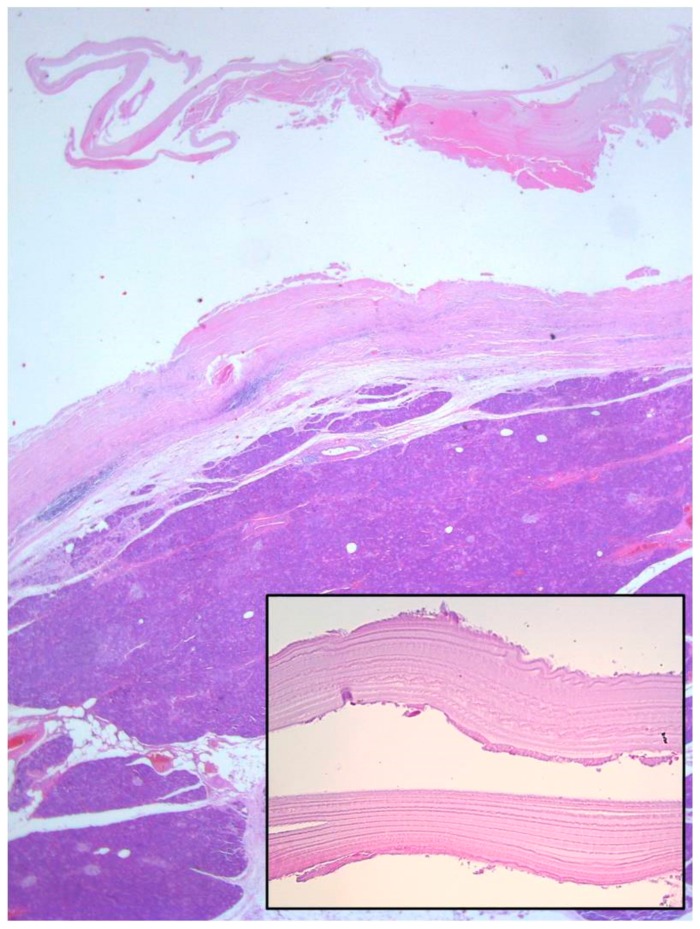
Histological examination of the pancreatic cystic lesion revealed a dense fibrous wall with hyalinization of the inner lining and uneven chronic inflammatory infiltration of the outer layer (Haematoxylin and eosin stain (H&E), 5×). On the inner surface and in the lumen, some hyalinized acellular layers were found, which is consistent with the laminated cuticle of the hydatid cyst, along with some hydatid sand. Parasitic scolices were not found (inlet, 40×).

The initial pathohistological examination of the specimen did not correspond to preoperative radiological findings highly suggestive of mucinous cystadenoma. Surprisingly, pancreatic hydatid disease with an ectocyst was found, with the fluid being positive for “hydatid sand”, scolices, and hooklets (Figure 6). Histological examination of the pancreatic cystic lesion revealed a dense fibrous wall with hyalinization of the inner lining and uneven chronic inflammatory infiltration of the outer layer. On the inner surface, and in the lumen, some hyalinized acellular layers were found, which is consistent with the laminated cuticle of the hydatid cyst, along with some hydatid sand. Parasitic scolices were not found (inlet) (Figure 7).

The postoperative course was entirely uneventful. On the seventh postoperative day, the patient was discharged from the hospital. The patient was subsequently examined every four months by abdominal ultrasound, and once a year by abdominal and pelvic CT. There has been no evidence of recurrence.

## 3. Discussion

Primary hydatid disease of the pancreas is an exceedingly rare phenomenon. Pancreatic infestation is mainly caused by hematogenous dissemination. The other possible ways in which the disease is spread are passage through the biliary system, peripancreatic lymphatic invasion, direct passage via the pancreatic veins, and retroperitoneal dissemination [1,6]. Pancreatic hydatid cysts (PHCs) are usually solitary (90–91%) and most often localized in the head of the pancreas. The tail of the pancreas is rarely affected [5]. The possible reason for the higher incidence of the pancreatic head involvement is its better vascularization [7].

Besides hydatid disease, *Ascaris lumbricoides*, *Clonorchis sinensis*, *Fasciola hepatica*, *Dicrocoelium dendriticum*, *Eurytrema pancreaticum*, Cryptosporidiidae, and Microsporidia can, in rare cases, cause pancreatic infestation [8].

Clinical presentation depends on the size of the cyst and anatomical localization. The main clinical symptoms are epigastric pain, weight loss, discomfort, and vomiting [9]. Cysts localized in the head of the pancreas can also cause obstructive jaundice due to external compression of the common bile duct [10,11]. Unusual complications of hydatid cysts involving the head of the pancreas are cholangitis, acute and chronic pancreatitis, duodenal stenosis or fistula, and pancreatic abscess [12,13]. Cysts in the pancreatic body and tail are usually asymptomatic and may be detected by the presence of an abdominal lump [1,6]. Rarely, cysts in the pancreatic tail can lead to splenomegaly, portal hypertension, rupture into the peritoneal cavity and gastrointestinal tract (GI) tract, or abscess formation [14].

The preoperative diagnosis of hydatid cysts is very difficult because the symptoms and the clinical findings may be similar to other, more commonly encountered cystic lesions of the pancreas [15]. The higher prevalence rate of pancreatic mucinous cystadenomas (2.6%), and on the contrary, the almost extraordinary occurrence of pancreatic echinococcosis leads to the fact that it is rarely considered in the differential diagnosis [16]. Ultrasound is an available, cost-effective and very sensitive method for the detection of internal cyst structures including membranes, septa, hydatid sand, and daughter cysts. It can show a well-defined anechoic lesion with a hyperechoic thick double-lined wall and internal echogenic material [17]. Endoscopic ultrasound has a higher diagnostic sensitivity than the transabdominal ultrasound (US) and the conventional CT scan, mainly for small lesions. Endoscopic ultrasound-guided aspiration provides biochemical and cytological analysis, which can exclude pancreatic pseudocysts [2]. On abdominal CT, the typical pancreatic hydatid cysts are multivesicular lesions with daughter cysts, hydatid sand, “water lily signs”, and a hyperdense cyst wall with calcifications [18].

During its evolution, the hydatid cyst undergoes significant structural and morphological changes. The radiological appearance of hydatid cysts may vary depending on the stage of the disease. Gharbi proposed the classification of hydatid cysts and estimation of their viability based on ultrasound appearance [19]. Simple cysts, e.g., Gharbi type I, do not demonstrate internal structures. Host response, degeneration, or decreasing intracystic pressure lead to detachment of the endocyst from the pericyst, so some kinds of membranes can be radiologically detected inside the cyst. Cyst wall degeneration along with daughter cysts appearing as cysts within a cyst, separated by the hydatid matrix (membranes, hydatid fluid, hydatid sand) of mixed echogenicity, can sometimes lead to findings very similar to mucinous cystadenoma. Most mucinous cystadenomas (MCNs) are oligolocular lesions, but unilocular appearance can also be seen [20]. The similarity to the detached membranes of an echinococcal cyst in such cases can be confusing. Considering radiological findings, our patient had a Gharbi type III cyst engaging the pancreatic body and tail. In favor of similarity, most MCNs are typically located in the body and the tail of the pancreas (93%) and in nearly all cases are found in women (>90%) [21].

Wall calcifications are also a common feature of both types of cysts. Although these are basically benign cysts, unlike a hydatid cyst where calcifications are almost common findings, the so-called “eggshell” calcifications in the MCN represent a "worrisome feature" and suggest a possible malignant transformation [22]. In our patient, these changes were interpreted as a strict indication for radical surgical treatment. Neither the echinococcal cyst nor the MCN should communicate with the main pancreatic duct. There are some cases reported in the literature where a hydatid cyst can cause an attack of acute pancreatitis as a result of prolonged cyst-induced compression and consequent development of cysto-pancreatic fistulization [22,23,24].

On MRI, pancreatic hydatid cysts are well-defined T2-weighted homogeneously hyperintense lesions with a hypointense rim and internal hypointense irregularities, which represent incipient detachment of the membranes [18]. Usually, typical radiological findings are not present, as in our case, so a hydatid cyst is an unexpected diagnosis. It is very difficult to differentiate PHCs from more common cystic lesions such as pseudocysts and benign or malignant pancreatic cystic neoplasms. PHC should always be considered in patients from endemic areas [1,6].

Radiological imaging (ultrasound and CT) and the epidemiological background of patients are the cornerstone for the diagnosis of abdominal hydatid disease. Because of significant morphological changes during hydatid cyst evolution, and the fact that it can affect almost any organ, a definitive diagnosis can be established only after histopathological confirmation.

Because of serious complications such as cyst suppuration, acute or chronic pancreatitis, jaundice, intraperitoneal rupture, or growing and spreading to surrounding organs, surgery is mandatory in every surgically eligible patient with pancreatic echinococcosis. As isolated pancreatic hydatid involvement is unusual, at present, there is still no agreement on an appropriate surgical approach. Basically, the surgeon has to decide between traditional pericystectomy and partial resection of the pancreas. There are two main surgical considerations: first, cyst localization, and second, the possible existence of a cysto-pancreatic fistula. One of the significant factors in the surgical decision is also the size of the cyst and the manner in which the cyst engages pancreatic tissue, or whether the cyst is manifesting mainly intraparenchymal or just a peripheral growth, engaging only a small part of the pancreatic tissue [25]. Management by hydatid cysto-pericystectomy (unroofing, fenestration) may be recommended in cases where the cyst is manifesting peripheral growth with a low probability of communication with the main pancreatic duct, or in cases where a cyst is adhering to the surrounding blood vessels or organs so dissection is estimated to be dangerous. The occurrence of a long-lasting pancreatic fistula can appear as a result of this method of treatment. In other cases, especially when the cysts affect the body or tail of the pancreas, pancreas resection is the surgical method commonly used [23].

According to the current recommendations, patients with a preoperative diagnosis of echinococcosis should always receive prophylactic anthelminthic agents (albendazole 10–15 mg/kg/day) for 2 to 4 weeks. This should continue for at least 4 weeks after surgery to reduce the risk of disease recurrence [26]. Chemotherapy is an effective therapy for younger patients, for small cysts with thin walls, for patients who are at high risk for surgery, for those with multiple peritoneal cysts, and to prevent secondary echinococcosis. It is also used as a concomitant therapy with percutaneous cyst drainage [27]. Percutaneous drainage of hydatid cysts (puncture, aspiration, injection, reaspiration—PAIR technique) is commonly used in the management of liver hydatid disease. When it comes to pancreatic echinococcosis, this method has yet to be tested. There is only one case reported in which percutaneous treatment and catheterization of a pancreatic hydatid cyst were successfully performed as an alternative to surgery [28].

In light of new perspectives on laparoscopic surgical management, in recent years, the interest in minimally invasive management of pancreatic diseases is rising. So far, two cases of successfully performed laparoscopic distal pancreatectomy for pancreatic hydatid disease have been published [1,29]. Our case, although initially planned as a treatment method for potentially malignant change, also confirms that laparoscopic distal pancreatectomy, with its numerous advantages over open surgery, can be a favorable therapeutic method for the management of pancreatic hydatid disease.

## 4. Conclusions

PHC is a rare phenomenon with a reported frequency of 0.1–2% of all cases of hydatid disease. The preoperative diagnosis of PHC is challenging because it can mimic a pseudocyst or cystic neoplasm of the pancreas. It should be considered in the differential diagnosis of cystic pancreatic lesions in patients from endemic regions.
